# Mechanisms of oxidative response during biodegradation of malathion by *S. oneidensis* MR-1

**DOI:** 10.1007/s11356-024-32283-4

**Published:** 2024-02-07

**Authors:** Qiaodong Pan, Yanhong Li, Jing Zhang, Ting Hu, Yu Hou, Shen Tang

**Affiliations:** 1 College of Environmental Science and Engineering, Guilin University of Technology, Jiangan Road 12, Guilin, 541004 Guangxi China; 2 Guangxi Key Laboratory of Environmental Pollution Control Theory and Technology, Guilin University of Technology, Guilin, 541004 China; 3 Collaborative Innovation Center for Water Pollution Control and Water Safety in Karst Area, Guilin University of Technology, Guilin, 541004 China

**Keywords:** *Shewanella spp*, Malathion, Oxidative stress, Biotransformation, Cellular characteristics

## Abstract

Malathion, an extensively used organophosphorus pesticide, poses a high potential risk of toxicity to humans and the environment. *Shewanella (S.) oneidensis* MR-1 has been proposed as a strain with excellent bioremediation capabilities, capable of efficiently removing a wide range of hard-to-degrade pollutants. However, the physiological and biochemical response of *S. oneidensis* MR-1 to malathion is unknown. Therefore, this study aimed to examine how *S. oneidensis* MR-1 responds physiologically and biochemically to malathion while also investigating the biodegradation properties of the pesticide. The results showed that the 7-day degradation rates of *S. oneidensis* MR-1 were 84.1, 91.6, and 94.0% at malathion concentrations of 10, 20, and 30 mg/L, respectively. As the concentration of malathion increased, superoxide dismutase and catalase activities were inhibited, leading to a significant rise in malondialdehyde content. This outcome can be attributed to the excessive production of reactive oxygen species (ROS) triggered by malathion stress. In addition, ROS production stimulates the secretion of soluble polysaccharides, which alleviates oxidative stress caused by malathion. Malathion-induced oxidative damage further exacerbated the changes in the cellular properties of *S. oneidensis* MR-1. During the initial stages of degradation, the cell density and total intracellular protein increased significantly with increasing malathion exposure. This can be attributed to the remarkable resistance of *S. oneidensis* MR-1 to malathion. Based on scanning electron microscopy observations, continuous exposure to contaminants led to a reduction in biomass and protein content, resulting in reduced cell activity and ultimately leading to cell rupture. In addition, this was accompanied by a decrease in Na^+^/K^+^- ATPase and Ca^2+^/Mg^2+^-ATPase levels, suggesting that malathion-mediated oxidative stress interfered with energy metabolism in *S. oneidensis* MR-1. The findings of this study provide new insights into the environmental risks associated with organophosphorus pesticides, specifically malathion, and their potential for bioremediation.

**Graphical Abstract:**

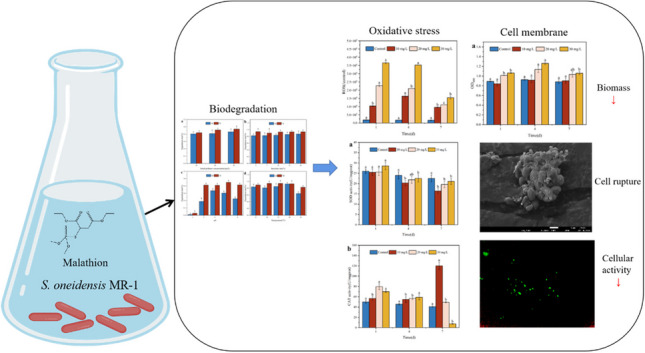

## Introduction

The increasing global population has resulted in a rising demand for food, leading to a greater reliance on pesticides for crop production. Pesticides encompass various organic chemical mixtures, such as insecticides, fungicides, and plant growth regulators. These compounds are crucial in several domains, including agriculture, aquaculture, horticulture, and home applications (Deng et al. 2018). Among the various types of pesticides, organophosphorus pesticides are the most widely used group (Zhao et al. 2021). Malathion (2-[(dimethoxythiophosphoryl)sulfonyl]butanedioic acid diethyl ester) is a non-systemic, broad-spectrum organophosphorus pesticide that can be used to control pests of trees, fruits, vegetables, and field crops, and in combating sanitary pests such as houseflies, mosquitoes, and parasites (Khan et al. 2016). Upon application onto agricultural land, pesticides are carried through runoff into aquatic ecosystems, leading to their persistent enrichment in groundwater (Tudi et al. 2021). The presence of malathion in the environment is of increasing concern because of its poor stability and tendency to mix with other components in water to form stable and more toxic intermediates, such as malathion, isomalathion, diethyl maleate, and dimethyl O, O-phosphate (Krstić et al. 2008; Vasseghian et al. 2022).

Malathion has a short degradation half-life; however, its high application rate has led to high detection rates for organophosphorus pesticides. Studies conducted in Jalisco, Mexico, indicated that malathion concentrations in rivers ranged from 311.8 to 717.3 μg/L, while in surface waters, the concentrations varied from 771.5 to 863.5 μg/L (Silva-Madera et al. 2021). Furthermore, monitoring the Sari Shahid Rajai Dam reservoir in Iran revealed that the average concentrations of organophosphorus pesticides were notably higher in spring and summer than in other seasons, with malathion reaching an average concentration of 0.8 μg/L in summer (Heidar et al. 2017). Meanwhile, malathion was found at 89.3 ng/L in surface water and 174.4 ng/L in groundwater in the North China Plain during summer in China (Wang et al. 2022). This could be attributed to the fact that spring and summer mark the onset of agricultural activities, which typically involve increased pesticide usage. As mentioned earlier, the prolonged and indiscriminate use of pesticides since their introduction in the USA in 1986 (Geed et al. 2016) has resulted in rapid contamination levels, posing a threat to the ecological environment and human health.

Most studies have confirmed the biological effects of malathion, including hepatotoxicity (Esen and Uysal 2018), pulmonary (Uysal and Karaman 2018), thyroid (Xiong et al. 2018), and reproductive and developmental toxicities (Bhardwaj et al. 2018). After a 15-day exposure, zebrafish brain tissue exhibited high concentrations of malathion (500 g/L), causing significant developmental toxicity in zebrafish embryos, including bradycardia, reduced hatching rates, malformations, causing abnormal movement patterns in zebrafish larvae (Cui et al. 2023). Malathion (0.001, 0.06, and 0.1 mg/L) also induces oxidative stress and antioxidant effects and even affects gene expression in the nerves of shaker mosquito larvae (Rebechi et al. 2021). In addition, malathion interferes with the activities of individual organisms and affects complex metabolic processes. Further, when malathion concentrations reach 150 or 300 mg/kg, it triggers a decrease in glutathione and paraoxonase activities in mice. Therefore, there is an upsurge in malondialdehyde (MDA) and nitric oxide concentrations, causing significant DNA damage and subsequent genotoxicity (Abdel-Salam Omar et al. 2018a). Excessive malathion exposure causes a range of adverse effects. For example, malathion exhibits cytotoxic properties and induces oxidative stress, leading to DNA damage and the destruction of pyrimidine bases (Olakkaran et al. 2020). Therefore, the toxic response of microorganisms to malathion degradation requires further investigation.

*Shewanella spp.* have garnered significant attention in recent years owing to their immense potential in environmental pollution management. *Shewanella spp.* are good candidates for remediation and detoxification because of their excellent electron donors and high metabolic capacity, which are essential for hard-to-degrade pollutants (Zou et al. 2018). It is well known that while toxic substances are used as substrates by microorganisms in biodegradation processes, they can also cause toxic reactions in microorganisms. Therefore, studying the oxidative response of *Shewanella spp.* to organophosphorus pesticides is essential. *S. oneidensis* MR-1 belongs to the genus *Shewanella* and is effective in degrading organic pollutants; for example, it can remove approximately 94.2% of sulfamethoxazole (Zhao et al. 2022). Previous studies have shown that *S. oneidensis* MR-1 possesses OxyR-regulated proteins involved in bacterial oxidative stress (Wan et al. 2017). However, the oxidative stress and cellular morphological changes associated with *S. oneidensis* MR-1 have not been thoroughly investigated.

Studies on *S. oneidensis* MR-1 biodegradation of organic matter provide valuable insights into the bacteria-contaminant relationship; however, there is a dearth of information regarding changes in bacterial biofilms during treatment. Specifically, there is a lack of data concerning changes in intracellular antioxidant enzymes within the organophosphorus pesticide degradation system of *S. oneidensis* MR-1. Therefore, this study aims to (1) investigate the biodegradation of malathion using *S. oneidensis* MR-1 as a model strain, focusing on four influencing factors as part of a laboratory-based theoretical study; (2) assess malathion-induced oxidative responses in *S. oneidensis* MR-1, including changes in antioxidant enzyme activity, malondialdehyde (MDA) content, and reactive oxygen species (ROS) production, providing evidence of oxidative stress; (3) identify changes in cellular properties under malathion stress and to analyze bacteriophage biomass, total intracellular protein, ATPase activity, and cell membrane integrity, serving as measures of cellular damage. The results of this study provide new insights into the toxicity and physiological and biochemical responses of malathion to bacteria.

## Materials and methods

### Chemicals and reagents

Malathion was purchased from Texas Green Bar Fine Chemical Co. Ltd. Yeast extract, tryptone, and sodium lactate were purchased from Sangong Biological Engineering Co. Ltd. Magnesium sulfate (MgSO_4_), potassium dihydrogen phosphate (KH_2_PO_4_), phosphate buffer solution (PBS), sodium chloride (NaCl), and other reagents were purchased from Xilong Science Co. ATPase, superoxide dismutase (SOD), catalase (CAT), total protein, and ROS assay kits were provided by the Nanjing Jiancheng Institute of Biological Engineering. All the chemicals were analytically pure.

Malathion masterbatch preparation: An appropriate amount of malathion emulsion (75%) was dissolved in methanol, and the concentration was adjusted to 2000 mg/L in a volumetric flask and stored in a refrigerator at 4 °C.

### Microbial culture and conditions

The selected test strain, *S. oneidensis* MR-1, is a Gram-negative bacterium purchased from the Marine Microbial Strain Conservation and Management Center of China (MCCC ATCC 700550). Luria–Bertani medium was prepared by dissolving tryptone (10 g/L), yeast extract (5 g/L), and NaCl (5 g/L) in distilled water. *S. oneidensis* MR-1 was cultured in a liquid medium and subjected to incubation at 30 °C for 24 h in a thermostatic blast oven (ZD-88, Jiangsu, China) until the logarithmic growth phase of the cells, preparing them for subsequent biodegradation experiments.

### Malathion biodegradation experiments

To investigate the factors influencing the degradation of malathion by *S. oneidensis* MR-1, the concentration of malathion stock solution was quantified after 24 h of pre-culturing *S. oneidensis* MR-1. To investigate the degradation of malathion by *S*. *oneidensis* MR-1, the following steps were followed: (1) In a sterilized 250-mL conical flask, add an inorganic salt culture solution (KH_2_PO_4_, K_2_HPO_4_, (NH_4_)_2_SO_4_, Na_2_SO_4_, CaCl_2_, MgSO_4_, and NaCl) and an appropriate amount of malathion solution. (2) Adjust malathion concentration to 10, 20, and 30 mg/L (to facilitate subsequent expression, we used 10 mg/L as the lower concentration, 20 mg/L as the medium concentration, and 30 mg/L as the higher concentration). (3) Inoculate the flask with *S. oneidensis* MR-1 at 1, 5, 10, 15, and 20% (v/v). (4) Adjust the pH to 4, 5, 6, 7, and 8 using 0.5 mol NaOH or HCl solution. (5) Set the shaker temperature to 25, 30, 35, 40, or 45 °C. (6) Ensure the total reaction volume is 100 mL. (7) Eliminate oxygen by passing sterile N_2_ gas. (8) Seal the flask with a film and place it in a constant temperature shaker (35 °C; 150 rpm). (9) Keep the malathion concentration fixed at 30 mg/L during the experiment, varying other conditions. (10) Regularly sample the reactions at specified intervals for measurement and analysis.

At the end of the biodegradation reaction, a certain amount of sample from 100 mL reaction solution was extracted with petroleum ether in equal proportions. The 3 mL extract was nitrogen dried and re-dissolved in 3 mL of methanol. The mixed extract was filtered through a 0.22-μm filter membrane. Malathion concentrations were determined by gas chromatography (GC) (6890N). The following GC conditions were used: FPD detector selected and set at 250 °C; column DB-35MS (30 m × 250 μm × 0.25 μm); inlet temperature: 200 °C, pressure: 11 psi; N_2_ flow rate: 6.5 mL/min, column chamber temperature: 150 °C, hold for 1 min; 10 °C/min to 200 °C, hold for 7 min; 5 °C/min to 260 °C, hold for 1 min; H_2_ flow rate: 100 mL/min, air flow rate: 130 mL/min, N_2_ flow rate: 30 mL/min. Three parallel experiments were conducted for each group to quantify the samples using the external standard method, and the averaged results were calculated.

### Oxidative stress measurement

The ROS assay was performed using a Nanjing Built Kit (E004-1–1 chemical fluorescence method). The test solution was transferred into a 10-mL sterilized centrifuge tube, centrifuge at 20 °C and 6000 rpm for 5 min, and the supernatant was discarded. Add 1 mL of PBS to the centrifuge tube containing the precipitate. Carefully re-suspend the precipitate by pipetting up and down. Transfer the re-suspended solution to a 1.5-mL centrifuge tube, centrifuge at 4 °C and 10,000 rpm for 5 min, and subsequently remove and discard the supernatant. No grinding was required for ROS determination. The total protein (TP) content of each bacterial sample was determined using the same kit to account for variations in bacteria quantities among the samples. Simultaneously, add 1 mL of sterile phosphate buffer solution, transfer the centrifuge tube to ice bath condition, and add 200 μL of zirconium oxide for grinding on a cell grinder (70 Hz, 10 min). A pipette gun was used to transfer 200 μL of supernatant to another 1.5-mL centrifuge tube, centrifuged for 5 min at 4 °C and 10,000 rpm, and stored at − 20 °C and in the dark. The supernatant was collected to assess CAT and SOD activities using an appropriate kit. The effect of malathion on ROS production in *S. oneidensis* MR-1 was evaluated by varying the malathion concentrations from 10 to 30 mg/L.

### Determination of MDA

The MDA content of the samples was determined using the thiobarbituric acid (TBA) method. A 0.2 g portion of the sample was taken and mixed with quartz sand, followed by adding 2 mL of 10% TCA (trichloroacetic acid), and ground until a homogeneous consistency was achieved. Subsequently, 8 mL of TCA was added, and the mixture was further grounded before being centrifuged at 10,000 rpm for 10 min. Following that, 2 mL of the centrifuged supernatant was added to 2 mL of TBA, mixed, allowed to react in a boiling water bath for 15 min, cooled rapidly, and centrifuged again. The absorbance of the supernatant was measured at 532, 600, and 450 nm, and the MDA and soluble polysaccharide contents of *S*. oneidensis MR-1 were calculated using Eqs. (1) and (2).

1 $$MDA\;\,\;concentration\;(\mu mol/L)=6.45 \times\;({A}_{532} - {A}_{600}) - 0.56{A}_{450}$$

2 $$Polysaccharide\;\,\;concentration\;(mmol/L)= 11.71{A}_{450}$$

### ATPase activity assay

Cells were collected in a liquid medium containing malathion at 1–7 days and washed twice with PBS for further experiments. The cells were first sonicated, and the supernatant was collected by centrifugation at 10,000 rpm for 15 min. The assay was performed according to the instructions of the kit manufacturer.

### Bioactive characteristics

#### Biomass measurement

The samples to be tested were washed two to three times with sterile 0.01 M PBS at pH 7.0. The samples were incubated in 0.1% crystalline violet solution for 10 min to stain live cells and washed twice with double-distilled water to remove unbound crystalline violet. Next, 1 mL of an ethanol-methanol mixture at a ratio of 8:2 was added and incubated for 1 h. Crystalline violet was absorbed by the elution. Finally, the cell density was quantified by measuring the absorbance at 595 nm using an ultraviolet–visible spectrophotometer. The effect of malathion on the growth of *S. oneidensis* MR-1 was determined by measuring the bacterial biomass.

#### Assay of cell activity

The samples to be tested were washed two to three times with sterile 0.01 M PBS at pH 7.0. For the cell fluorescence-imaging test, both the experimental and control cells were treated with PI dye and SYTO 9 to stain the live cell nuclei. The fluorescence signal of the bacteria was then observed under excitation wavelengths of 535 and 485 nm. This is to test the effect of the contaminants on the integrity of the cell membrane of *S. oneidensis* MR-1.

#### SEM measurement

Bacteria were centrifuged (6000 rpm; 3 min) after 7 days of exposure to malathion at a concentration of 30 mg/L and then washed three times with PBS solution. Subsequently, the bacteria were fixed overnight in a 2.5% glutaraldehyde solution at 4 °C. After removing the fixative, the samples were washed with PBS solution three times for 15 min each and then dehydrated with ethanol at concentrations of 30, 50, 70, 90, and 100% for 15 min each, followed by freeze drying for 8 h. The bacteriophage powder was glued to the sample table using conductive glue and sprayed with gold. The samples were observed using SEM (JSM-6380LV, Japan).

### Statistical analysis

All experimental results were presented as three replications and data were analyzed using standard error. The SPSS 26.0 software package was used for statistical analysis. Data on the degradation experiment as well as physiological and biochemical indices in *S. oneidensis* MR-1 were subjected to one-way analysis of variance (ANOVA) with *p* < 0.05 by Duncan’s test to determine the significance of the differences between treatment groups.

## Results and discussion

### S. oneidensis MR-1 biodegradable malathion

**Fig. 1:**
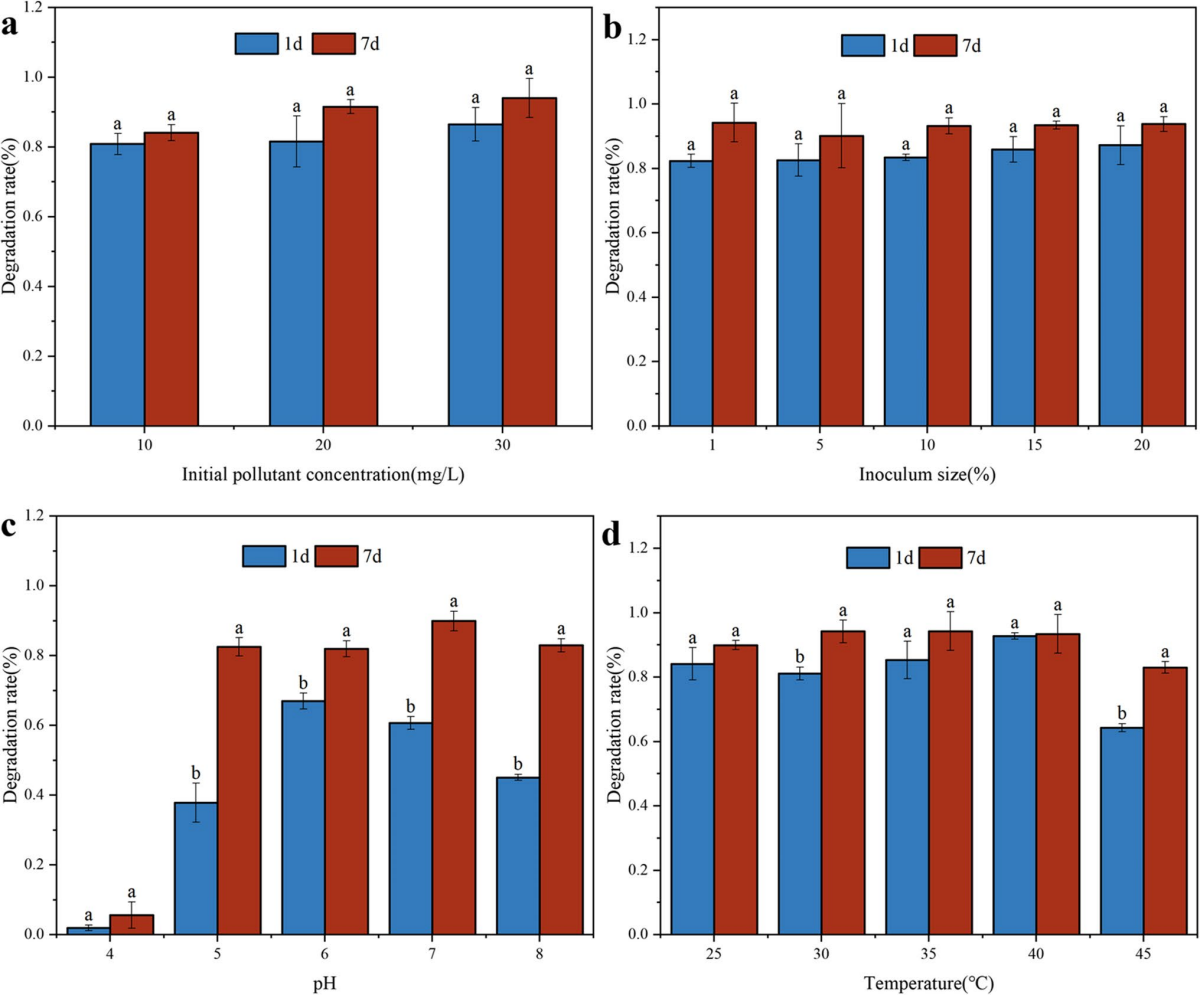
Characterization of malathion degradation by *S. oneidensis* MR-1. **a** Effect of initial concentration of malathion; **b** effect of bacterial inoculum levels; **c** effect of pH; **d** effect of temperature

Figure 1a illustrates the biodegradation of *S. oneidensis* MR-1 by different concentrations of malathion. When the initial concentration was 10 mg/L, 84.1% of malathion was removed within 7 days. Malathion dissipated more rapidly as the pollutant concentration increased to 20–30 mg/L, reaching a maximum removal of 94.0% on day 7. The high biodegradability of *S. oneidensis* MR-1 exhibited at different malathion concentrations can be attributed to its high tolerance to organophosphorus pesticides. Other strains, such as *B. safensis* strain FO-36b^T^, *Bacillus subtilis* KCTC 13429^ T^, and *Bacillus subspecies* in aquosorum strain KCTC 13429^ T^, isolated from Sudanese pesticide-contaminated soil, showed good pesticide biodegradation efficiency, achieving over 80% removal of 400 mg/L malathion in 60 days (Ishag et al. 2016). Dar et al. (2022) used the micrococcus MAGK3 obtained from Astragalusglaucus agricultural soil to degrade the 1000 μL/L malathion approximately 100% in 360 h. Researchers have confirmed that this strain can biodegrade malathion, using it as the sole carbon and energy source. Furthermore, it is hypothesized that appropriate concentrations of pesticide-induced growth of *S. oneidensis* MR-1 may promote the degradation of organophosphorus pesticides, thereby increasing the removal efficiency of malathion from the medium.

The biodegradation of organic pollutants is achieved by converting organic pollutants through the injection of microorganisms. However, the concentration of the inoculum is not standardized, and the quantity and quality of bacteria directly affect biodegradation. Therefore, the malathion biodegradation was examined at different inoculation ratios (Fig. 1b). At the beginning of degradation, in the medium containing 30 mg/L malathion, the degree of degradation gradually increased with an increasing percentage of inoculation. However, it was observed that over 90% of the organophosphorus pesticides in all five inoculum batches were degraded by day 7 after the addition of malathion. The highest degradation efficiency was achieved with an inoculum size of 1%. Similar results indicate the degradation of toxic and hazardous organic phenols by *Bacillus* CYR2 is strongly influenced by the inoculum level, with alkylphenols removed from the medium as the inoculum level increases from 2 to 4% (Reddy et al. 2017), which is similar to the results of this study. In addition, the inoculum concentration in the system was increased by the combined colonies for better degradation, and the mixture can be used for the synergistic biodegradation of aromatic-aliphatic co-polyester plastics. Simultaneously, bacteria use pollutants as their sole carbon source to survive (Meyer-Cifuentes et al. 2020). Therefore, a suitable inoculum size should be selected for the biodegradation process to avoid competition for resources between bacteria owing to excessive inoculum concentrations, which can lead to inefficient degradation.

Figure 1c illustrates the effect of pH on malathion biodegradation by *S. oneidensis* MR-1. The degradation rate of 30 mg/L of malathion increased with increasing pH. However, at pH 7, the lowest amount of malathion remained in the medium during the later stages of degradation, with a degradation rate of 90%. Notably, the early stages of degradation at pH 6 were significantly more effective than in other groups. The results showed that *S. oneidensis* MR-1 is most effective at removing malathion under neutral pH conditions. This may be because neutral conditions are more favorable for bacterial growth and promote the synthesis and secretion of biofilms and associated enzymes (Li et al. 2023). In addition, researchers discovered that *S. oneidensis* MR-1 exhibited optimal degradation efficiency under neutral conditions when breaking down organic pollutants, such as metal complex azo dyes (Li et al. 2021).

Since the incubation temperature affects bacterial metabolism and enzymatic activity, we examined how different temperatures influenced the biodegradation of malathion by *S. oneidensis* MR-1. As shown in Fig. 1d, comparing the degradation rates before and after the process revealed that the highest degradation rates were observed at 35 °C. Higher degradation rates were also observed at 25, 30, and 40 °C. The degradation rate decreased slightly (83%) when the temperature was increased to 45 °C. The decrease could be attributed to the influence of temperature on the activity of relevant enzymes. Extreme temperature, whether too high or low, can result in a loss of bacterial activity (Xiao et al. 2012), thus, affecting the efficiency of biodegradation. Similar findings were obtained in a study by Liu et al. (2011), where the biocatalytic activity of *S. oneidensis* MR-1 biofilms first increased and then decreased as the temperature increased from 30 to 45 °C owing to the inactivation of bacterial metabolism or changes in physiological behavior.

### Oxidative response of S. oneidensis MR-1 under malathion stress

#### Malathion-induced reactive oxygen species (ROS) production in S. oneidensis MR-1 cells

**Fig. 2:**
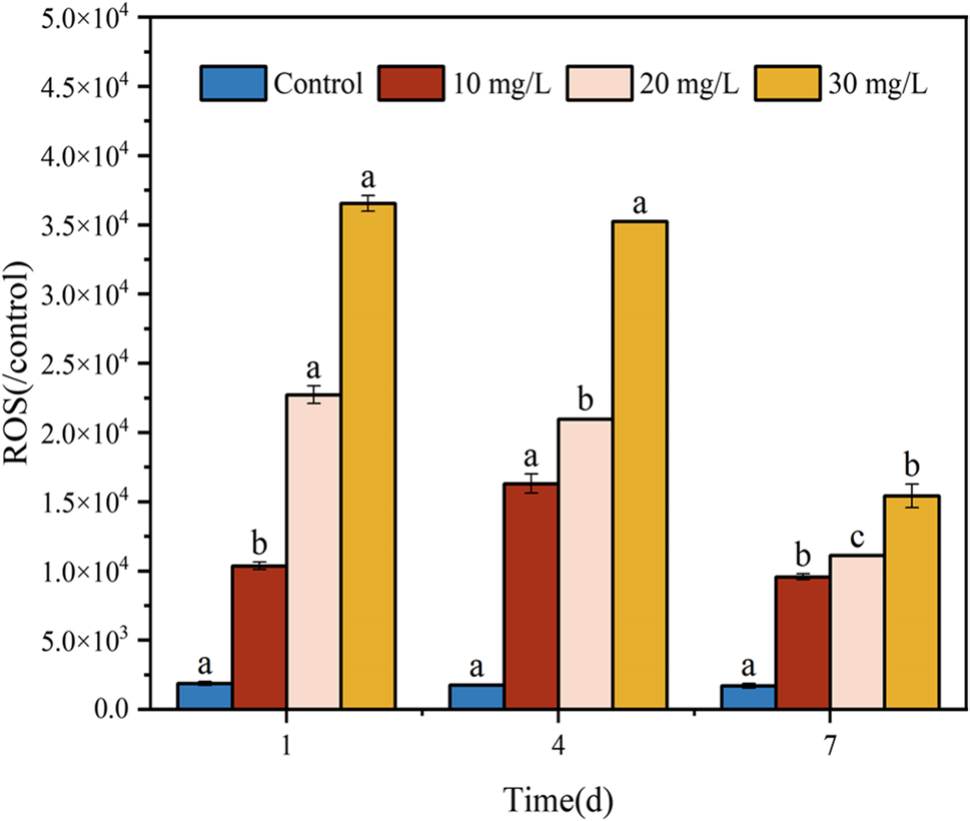
Malathion-induced changes in intracellular ROS levels

ROS, a byproduct continuously generated during aerobic respiration, maintains redox homeostatic signaling and plays a crucial role in regulating signal transduction, gene expression, and functional cellular responses under physiological conditions (Angel Torres 2010). There exists a delicate balance between the production and breakdown of intracellular ROS levels. However, when organisms are exposed to adverse environmental factors, ROS levels surge, leading to membrane damage, dysfunction of specific proteins, and metabolic impairment of biochemical processes (Zhang et al. 2019; Fasnacht and Polacek 2021). At low concentrations (10 mg/L), there was no change significantly in ROS levels than in the blank group, probably because of the high resistance of *S. oneidensis* MR-1 to malathion. Figure 2 shows the results. However, as the dose of malathion increased > 20 mg/L, intracellular ROS levels were 21.4% and 121.6% higher than those in the control group, indicating that malathion significantly elevated ROS levels in the bacteria. Zhao et al. (2015) have demonstrated that short-term exposure of *Carassius auratus* cells to the organophosphorus pesticide monocrotophos produces ROS that are difficult to clear, similar to the present study. This suggests that the key events associated with ROS overload can be divided into two categories. First, the target contaminants introduced to the culture can directly react with the small molecules within the cells. However, excessive ROS levels are accompanied by the disruption of the normal function of the antioxidant system and, consequently, a loss of regulatory capacity, leading to apoptosis (Ren et al. 2016; Hasanuzzaman et al. 2021). Notably, malathion instability results in the biotransformation of *S. oneidensis* MR-1 into malaoxon (Aker et al. 2008). It has been established that this oxidation product is more toxic than the original malathion compound (Meng et al. 2020) and may further stimulate the bacterium to trigger ROS bursts, which may contribute to the accumulation of ROS in *S. oneidensis* MR-1 cells.

### Malathion-induced changes in S. oneidensis MR-1 SOD and CAT activities

Activating the antioxidant system is a well-known defense mechanism in organisms against damage caused by oxidation, a primary and universal detoxification mechanism (Yang et al. 2016). Antioxidant levels are regulated through SOD and catalase CAT synthesis, which acts as an adaptation to severe physiological and biochemical disturbances triggered by oxidative stress. SOD and CAT play essential roles in the timely elimination of ROS. SOD primarily converts O_2_^−^ to O_2_ and H_2_O_2_, while CAT is usually responsible for the further degradation of H_2_O_2_ into non-toxic H_2_O and O_2_ (Jiang et al. 2015).

**Fig. 3:**
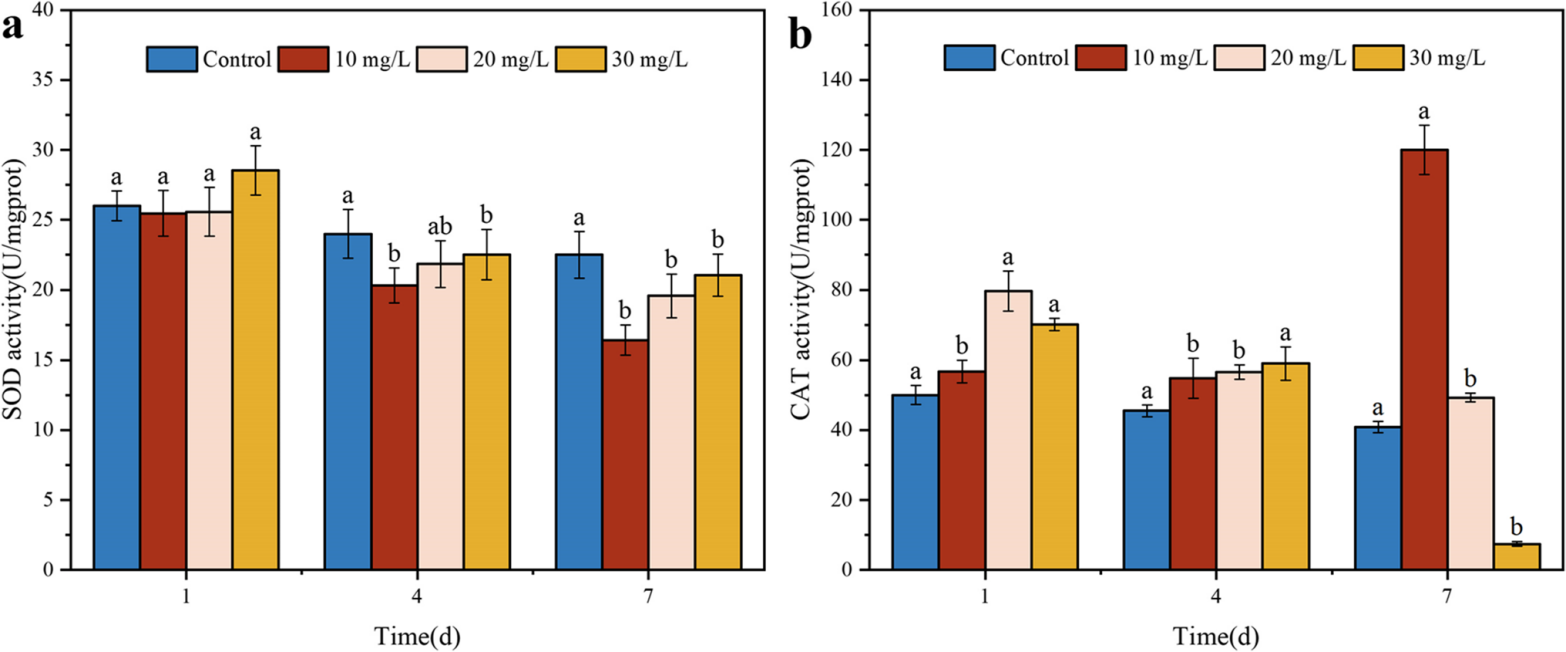
Effect of initial concentration of malathion on the level of SOD and CAT activity in *S. oneidensis* MR-1 treated with (**a**, **b**)

Figure 3 shows the changes in the SOD and CAT activities in response to malathion in each dose group of *S. oneidensis* MR-1. SOD activity exhibited an overall decreasing trend after 1–7 days of treatment with varying doses of malathion (Fig. 3a). SOD levels increased as malathion concentration increased from 10 to 30 mg/L. A similar trend was observed in Fig. 3b. In contrast to the low-content treatment (10 mg/L) at 7 days, CAT levels showed a substantial increase of 65.9% than in control. The changes in SOD and CAT activity in *S. oneidensis* MR-1 under malathion-induced stress followed almost a similar pattern, suggesting potential synergistic effects of both antioxidants in resisting oxidative stress. In a short-term toxicity assay of triazophos on *Carassius auratus*, SOD and CAT activities were decreased in goldfish (Liu et al. 2015), which is consistent with our situation. In contrast, SOD and CAT activities were significantly elevated when *Nitzschia palea* was present in the presence of trichlorfon and acephate (Wang et al. 2020). It is inferred that SOD is initially triggered to activate the breakdown of O_2_^−^ while promoting CAT activity to convert H_2_O_2_ to H_2_O and O_2_. Previous studies showed that SOD and CAT contents of oily microalgae increased after exposure to malathion during degradation, suggesting that antioxidant enzymes can catalyze the removal of free radicals and protect bacteria from oxidative stress (Nanda et al. 2019). In addition, malathion exposure induces changes in the intracellular antioxidant enzymes of *Anabaena variabilis*, including an increase in the levels and expression of SOD and CAT (Ningthoujam et al. 2013). Exposure of goat precursor follicles to high concentrations of malathion resulted in increased CAT and SOD activities, leading to the loss of membrane integrity and accelerated apoptosis (Bhardwaj and Saraf 2016). The findings align with the observed increase in ROS levels in the “Malathion-induced reactive oxygen species (ROS) production in *S. oneidensis* MR-1 cells” section and suggest that higher content exposures can cause associated tissue damage or breakdown due to greater stress mechanisms exerted on the antioxidant system.

### Malathion-induced changes in MDA content and soluble polysaccharide production in S. oneidensis MR-1

**Fig. 4:**
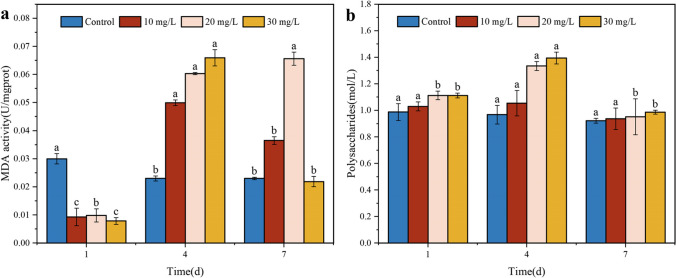
Effect of malathion treatment on the level of MDA activity (**a**) and polysaccharide content (**b**) of *S. oneidensis* MR-1

**Fig. 5:**
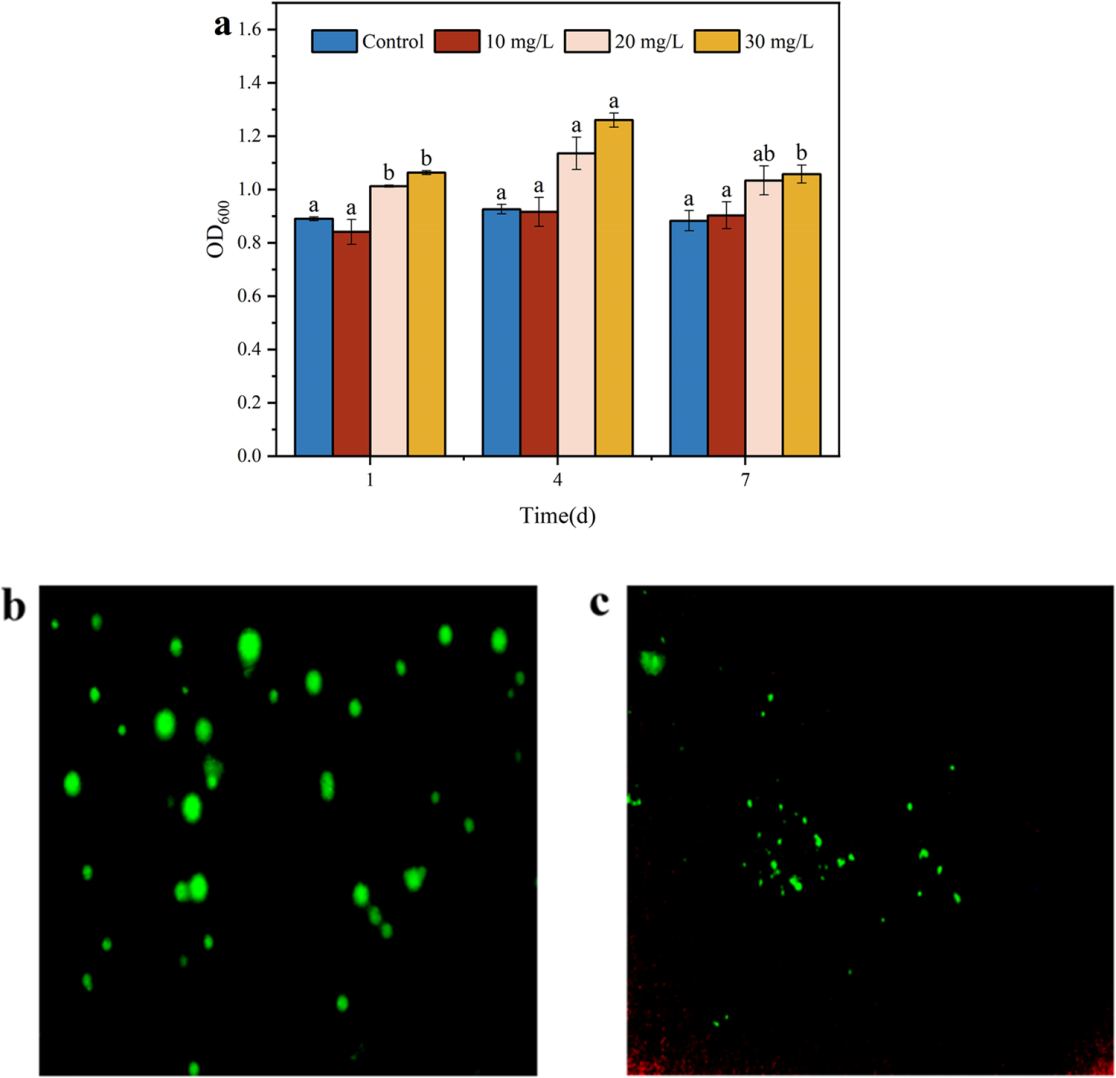
Effect of malathion on bacterial biomass (**a**) and cellular activity **b** before and **c** after degradation

MDA is a recognized marker of lipid peroxidation and is widely used to assess the degree of oxidative damage caused by environmental stresses (Ojha et al. 2011). The effect of stress on MDA content at variation initial pollutant concentrations was examined. As shown in Fig. 4a, MDA levels did not exhibit a significant change than in the control at 1 day. However, after 4 days, the malathion concentration increased from 10 to 30 mg/L, followed by an overall decreasing trend. MDA levels did not change significantly at 1 day compared to the control; however, after 4 days, as the malathion concentration increased from 10 to 30 mg/L, MDA levels peaked and subsequently showed an overall decreasing trend (Fig. 5a). This phenomenon can be attributed to the initial positive response to the target pollutant during the initial exposure stage, partially mitigating the damage caused by oxidative stress in *S. oneidensis* MR-1. Tao et al. (2020) also found an increase in MDA content of *Chlorella pyrenoidosa* in a mixture of three organophosphorus pesticides. It has been reported that prolonged exposure of cells to excessive amounts of malathion, as one of the most toxic insecticides to the liver and nerves, may overwhelm the antioxidant system inadequate to eliminate free radicals (Abdel-Salam et al. 2018b). Further, prolonged exposure of cells to excessive amounts of malathion may overwhelm the antioxidant system inadequate to eliminate free radicals. This is evidenced by the decrease in SOD content and CAT activity of *S. oneidensis* MR-1, particularly after the exposure time extended beyond 4 days. Consequently, the strain was unable to respond positively and promptly to a large accumulation of ROS, ultimately causing lipid peroxidation. Based on existing evidence, ROS can react with unsaturated fatty acids and cytoplasmic macromolecules, producing lipid oxidation, resulting in the damage of cells (Shi et al. 2009). In addition, in carp exposure to malathion, there is an observed increase in intracellular MDA content, accompanied by a significant decrease in the levels of antioxidant enzymes, including SOD, CAT, and GPx (Nasirin et al. 2023). These findings correspond to an increase in ROS levels.

Polysaccharides possess good antioxidant activity, which protects the body against oxidative stress by increasing the activity of antioxidant enzymes while reducing the MDA content (Chen et al. 2021). Therefore, we determined the polysaccharide content of *S. oneidensis* MR-1 following exposure to malathion. In the reaction system with malathion concentration ranging from 10 to 30 mg/L, *S. oneidensis* MR-1 exhibited similar polysaccharide content to that of the control, indicating a lack of significant oxidative stress (Fig. 4b). However, as the exposure duration increased, the polysaccharide content increase in all malathion-exposed groups showed a rise on day 4, followed by a return to the control level. The increase in soluble polysaccharide content was positively correlated with the malathion concentration. Rhizobia have been reported to secrete sticky substances, primarily composed of extracellular polysaccharides, to adapt to the harsh environment (Kopycinska et al. 2018). These findings are consistent with those reported in the literature. Furthermore, there is a correlation between the production of extracellular polysaccharides and ROS levels, thus confirming that the surge in extracellular polysaccharides is a direct response to ROS (Han et al. 2019). Combining the results of SOD and CAT, it is evident that *S. oneidensis* MR-1 activates its antioxidant system in response to malathion exposure and that the high secretion of polysaccharides was attributed to resistance to oxidative stress. However, when the release of ROS irreversibly damages the antioxidant defense and detoxification system, *S. oneidensis* MR-1 shows a series of adverse effects, such as respiratory damage, cellular ion imbalance, and regulatory disorders, leading to failure of the protective effect of the polysaccharide (Tian et al. 2023).

### Changes in the cellular characteristics of S. oneidensis MR-1 under malathion exposure

#### Effect of malathion on bacterial biomass

The toxic effect of malathion on *S. oneidensis* MR-1 cell growth exhibited a gradual increase as the malathion concentration increased from 10 to 30 mg/L (Fig. 5a). Further, when the malathion concentration was 10 mg/L, and the exposure duration was 4 day, there was no significant alteration in *S. oneidensis* MR-1 cell density compared to the blank group. However, applying malathion at 20–30 mg/L concentrations increased cell density by 13.8–18.1% and 19.4–23.9%, respectively. As the exposure time increased from 4 to 7 days, a general decrease in bacterial biomass was observed, suggesting that malathion can affect *S. oneidensis* MR-1 metabolism. At lower concentrations (10 mg/L) of malathion, the proteins secreted by the bacteria and the metabolites produced within 4 days seem to alleviate stress. Cyanobacterium *Scytonema* sp. BHUS-5 showed an increase in biomass in the presence of methyl parathion (Tiwari et al. 2017), a result that coincides with the findings of the above study. Malathion has been reported to serve as the sole carbon, phosphorus, and sulfur source for bacteria and fungi in the environment (Kumar et al. 2018). Therefore, *S. oneidensis* MR-1 may directly and indirectly use malathion for cellular self-propagation, potentially in the presence of malathion. However, after 4 days, the bacteria continued to resist oxidative stress, decreasing *S. oneidensis* MR-1 biomass. Specifically, the biomass in all malathion-treated groups remained higher than in the control group. This indicated that *S. oneidensis* MR-1 exhibited high resistance to malathion, implying that the cell viability of the bacteria was not inhibited in the presence of malathion.

To further investigate the changes in *S. oneidensis* MR-1 during malathion biodegradation, fluorescence images of *S. oneidensis* MR-1 were captured following treatment with SYTO 9 and propidium iodide at a malathion concentration of 30 mg/L (Fig. 2b, c). Typically, when the plasma membrane remains intact, propidium iodide cannot directly penetrate the interior of the cell. However, if there are breaches in the membrane, propidium iodide can smoothly enter the cell and generate red fluorescence with intracellular macromolecules, such as DNA and RNA. In contrast, the SYTO 9 can cross the cell membrane and stain the cells green. Higher bacterial activity is indicated by a more intense green fluorescence (Springthorpe et al. 2019; Bouchelaghem et al. 2022). SYTO 9 and propidium iodide staining experiments showed that 30 mg/L malathion caused less damage to *S. oneidensis* MR-1 cell membranes in the early stages of degradation. As the duration extended to 7 days, a decrease in green fluorescence coincided with an increase in red fluorescence, which is consistent with the results for biomass. This confirms that the cellular defense system can promptly repair the stress response induced in the system during the pre-exposure period. This further enhances the tolerance and accelerates the proliferation or differentiation of the cells (Huang et al. 2018), indicating the excellent biodegradability of *S. oneidensis* MR-1.

### Effect of malathion stress on the ATP activity of S. oneidensis MR-1

**Fig. 6:**
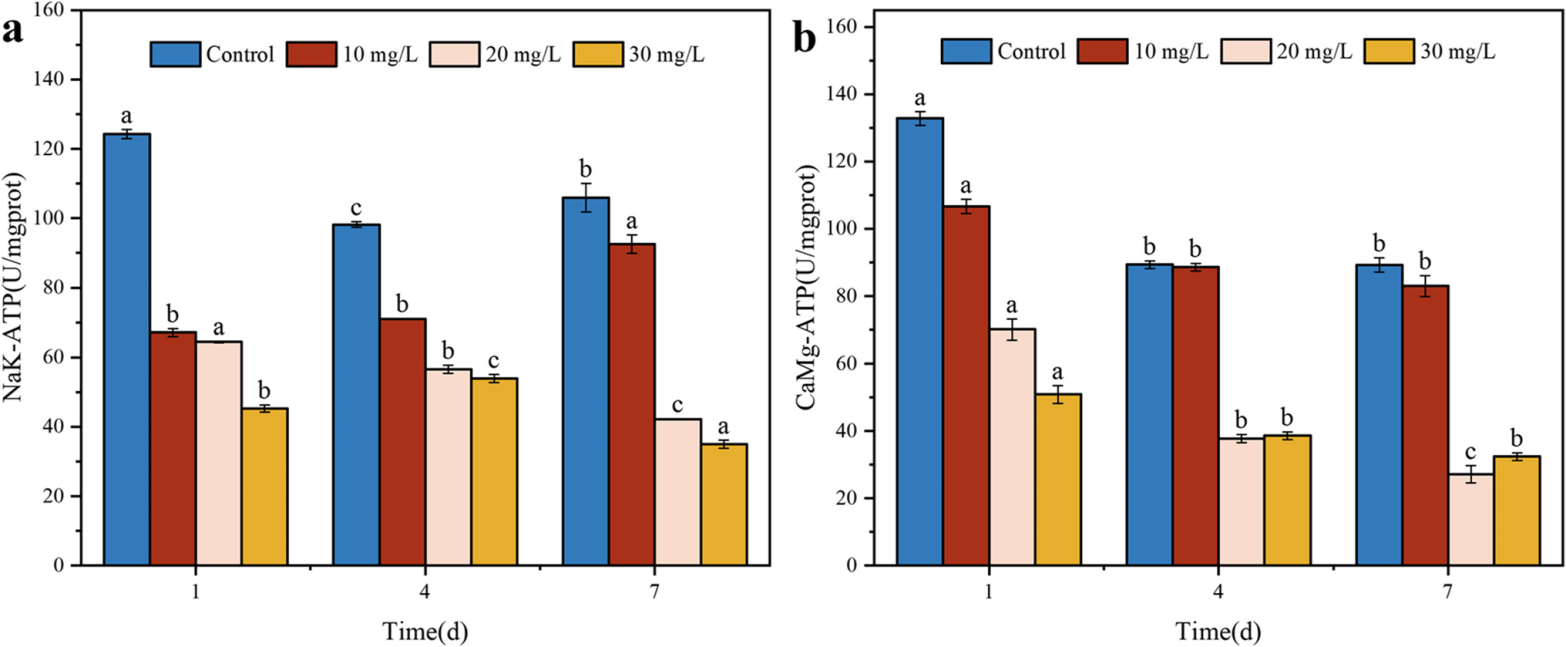
Effect of malathion exposure on *S. oneidensis* MR-1 Na^+^/K^+^- ATPase (**a**) and Ca^2+^/Mg^2+^- ATPase (**b**) levels

ATPases are vital cellular enzymes that facilitate different biological functions of cells, such as maintaining osmotic pressure, transporting ions across membranes, and regulating signal transduction. They also provide essential nutrients to cells and participate in cell energy metabolism, thereby influencing cell growth and reproduction (Ji et al. 2009). Further, when cell membranes suffer damage, harmful contaminants can disrupt ATPase interactions or interfere with energy metabolism pathways, ultimately causing changes in ATP levels within the organism (Poopal et al. 2013). The effect of malathion on this bacterium was examined by measuring the activities of Na^+^/K^+^-ATPase and Ca^2+^/Mg^2+^-ATPase. Figure 6 shows the results. The activities of Na^+^/K^+^-ATPase and Ca^2+^/Mg^2+^-ATPase exhibited a significant decrease following malathion treatment at concentrations of 10, 20, and 30 mg/L compared to controls. The changes ranged from 46.1 to 63.6% and 19.7 to 61.7%, respectively (Fig. 6a and b). This finding indicates that malathion stress inhibited ATPase activity. A study by Osman et al. (2021) also found that chlorpyrifos caused a significant decrease in ATPase levels in male rats. This inhibition may be attributed to the hydrophilic properties of organophosphorus pesticides, whereby malathion can bind to the active center of ATPase and inhibit ATP hydrolysis, subsequently reducing intracellular ATP synthesis and utilization (Coremen et al. 2022). However, Na^+^/K^+^-ATPase activity was significantly higher when exposed to low doses of malathion, suggesting that moderate concentrations of malathion exert an inductive effect on ATPase. This may be linked to the energy demand of *S. oneidensis* MR-1 for ATP hydrolysis, which accelerates the translocation and clearance of malathion, thereby triggering an increase in ATPase activity. Furthermore, an increase in the ATPase activity was also observed during the degradation of BDE-47 by *Pseudomonas aeruginosa* (Tang et al. 2016). Hence, when considering the role of ATPases, it can be inferred that inhibiting bacterial growth and malathion removal by high pollutant concentrations is associated with the decline in *S. oneidensis* MR-1 ATPase activity, disrupting energy metabolism.

### Effect of malathion on intracellular total protein content of S. oneidensis MR-1

**Fig. 7:**
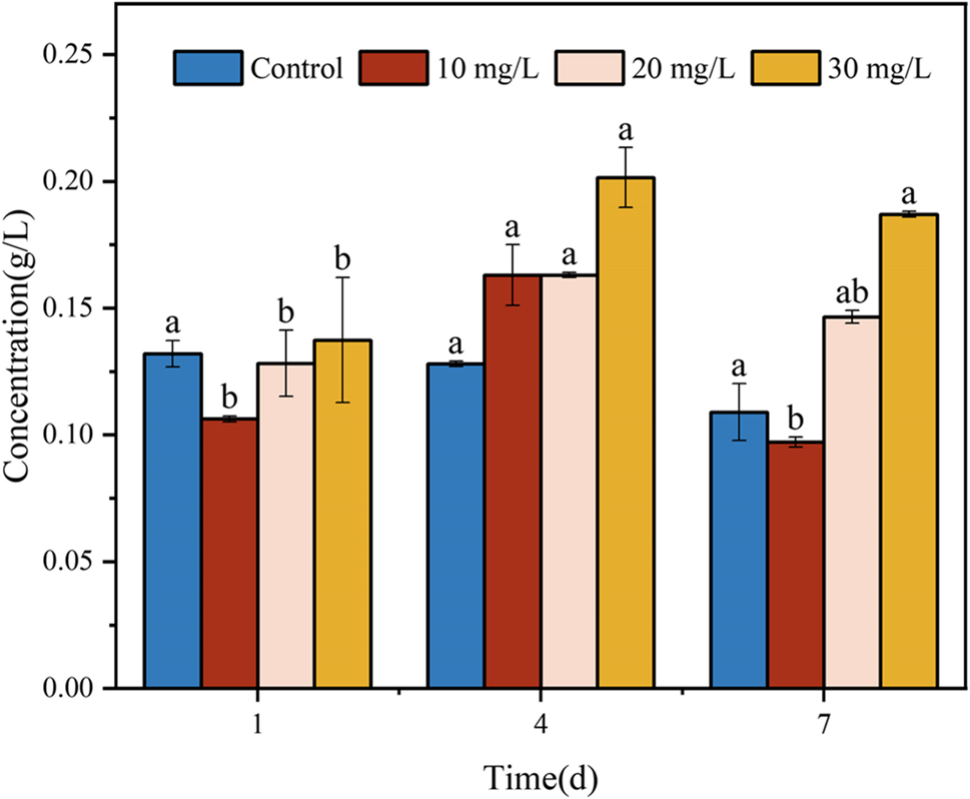
Effect of malathion exposure on the total protein content of *S. oneidensis* MR-1

Protein content serves as an auxiliary indicator for monitoring cell growth and DNA damage and is susceptible to oxidative stress caused by free radicals and other oxidants inside and outside the cell (Pattarachotanant and Tencomnao 2020). Therefore, the changes in the intracellular protein levels of *S. oneidensis* MR-1 during malathion exposure were examined. Figure 7 shows the results. There was a significant increase in intracellular protein concentrations from day 1 to 4 as malathion concentrations rose from 10 to 30 mg/L. This finding is consistent with those of Ibrahim et al. (2014), who observed a similar significant rise in protein content in *Spirulina obtusifolia* at lower concentrations of malathion (0.2 and 20 mg/L) stress. This phenomenon can be attributed to the oxidative response triggered by the target contaminant, suggesting that strain proteins play a protective role. This finding implies that appropriate oxidative stress can stimulate the organism to develop an enhanced antioxidant capacity. Specifically, the levels of intracellular protein content exhibited an initial increase followed by a decrease in the systems exposed to different doses of malathion. The toxicity of malathion impedes protein synthesis and downregulates the expression of certain proteins (Venkatesan et al. 2017; Amin et al. 2022). Moreover, the oxidative stress triggered by malathion causes protein oxidation, aggregation, and fragmentation, thereby altering the structure and function of proteins (Flores et al. 2017). In addition, exposure to malathion produces hepatotoxic effects in South Asian pangolins, as evidenced by a decrease in protein content over time and a significant increase in ROS, LPO, and DNA damage (Ullah et al. 2018). Based on the above findings, *S. oneidensis* MR-1 intracellular proteins are crucial in combating oxidative reactions. Nevertheless, prolonged exposure to malathion-rich systems intensifies protein-damaging effect of malathion, leading to a reduction in intracellular protein content at later stages of exposure.

### Effect of malathion stress on the morphology of S. oneidensis MR-1

**Fig. 8:**
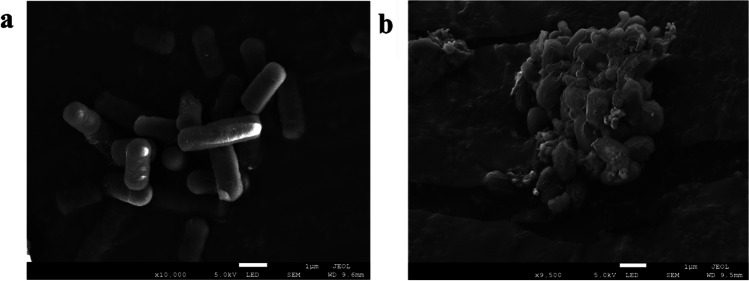
SEM of *S. oneidensis* MR-1 before (**a**) and after (**b**) exposure on malathion

Shahid et al. (2021) found that exposure of microorganisms to pesticides and other hazardous pollutants can alter their cellular morphology or even lead to cell destruction. In this study, SEM was used to investigate how the morphology of *S. oneidensis* MR-1 was affected by exposure to 30 mg/L malathion, before and after exposure. *S. oneidensis* MR-1 cells in their normal state were smooth, short-rod shaped, with a relatively uniform dispersion in the system (Fig. 8a). Following the late exposure of *S. oneidensis* MR-1 to 30 mg/L malathion (Fig. 8b), almost all bacterial cells showed irregular folds on their surface. This alteration in morphology is potentially attributed to oxidative stress-induced loss of intracellular material. These findings elucidate the reasons behind the observed decrease in bacterial activity, leading to the inactivation of relevant enzymes and a decrease in protein content at later stages of exposure.

## Conclusion

Our findings show that *S. oneidensis* MR-1 exhibits remarkable degradation capacity, achieving a removal efficiency of > 90% for malathion at the end of degradation under various conditions. We also found that different concentrations of malathion had harmful effects on *S. oneidensis* MR-1 bacteria. Specifically, short-term exposure to malathion leads to the activation of the antioxidant system, reducing SOD and CAT enzyme activities, increasing MDA content, and secretion of soluble polysaccharides. These responses were attributed to a direct reaction to the ROS produced within the cells. However, prolonged treatment with malathion causes bacterial inactivation, resulting in lipid peroxidation and changes in cellular properties. In addition, when *S. oneidensis* MR-1 is exposed to malathion, they exhibit an increase in intracellular ROS levels, reduced ATPase and cellular activities, and cell rupture with ruffling. Simultaneously, the pre-pollutant-induced oxidation reaction stimulated significant protein production. However, prolonged malathion exposure to the system inhibits intracellular protein synthesis in the strain. These findings highlight the association between oxidative cell damage and oxidative stress induced by the accumulation of high levels of ROS. Evidence suggests that *S. oneidensis* MR-1 can use malathion as a carbon source to alleviate the toxicity of its intermediate products. However, further investigation is needed to elucidate the changes in mitochondria, DNA, and intracellular ions due to apoptotic mechanisms. Physiological changes serve as early indicators of the cellular response to oxidative stress, and cells employ various mechanisms to prevent injury. The findings of this study can serve as a useful reference for understanding the physiological responses to bacterial biodegradation of environmental toxicants.

## Data Availability

Data available on request from the authors. The data that support the findings of this study are available from the corresponding author, Shen Tang, upon reasonable request.
